# Modern Sociology

**Published:** 1896-11-07

**Authors:** 


### Modern Sociology.

### MISTAKEN IMPRESSIONS OF PRISON LIFE.—I.

Various circumstances have of late drawn the attention of the public strongly to the details of prison life, with the result that the fact of many mistaken impressions existing popularly on that subject has been clearly brought to light. A paper published by the Howard Association, and entitled, "Charles Dickens' Prison Fictions," shows that serious evils may be produced by such false ideas. They have greatly hindered the efforts made in America to enforce the wise treatment of prisoners, especially as regards the separate system.

The secretary of the Howard Association has dealt judiciously with this special subject in a long letter to the *Times*, and it is one on which, perhaps more than most others, the greatest misconception exists. Unhappily, the false impressions obtaining on that point, as well as on prison discipline generally, have been greatly increased by the sensational statements put forth in the papers as regards the dynamitards recently liberated before the expiration of their term of sentence. There appeared also some months ago in a high-class periodical an able article on professional crime, dealing with the whole question as to the duty of the State towards criminals. The paper is very valuable from the manner in which it ventilates many points in the right administration of justice, which undoubtedly require the attention of the Legislature, and we shall have occasion to refer to it later in detail. We must first, however, endeavour to elucidate the nature of the mistaken idea existing almost universally on several important features of prison discipline. These are mainly due to the fact that the whole subject is viewed from outside the prison walls by those who have no opportunity of watching from day to day the real influence of the hidden life in these receptacles for offenders against the law. This is eminently the case with the thoughtful writer of the important paper we have referred to. A matter which affects the existence and the health, mental and physical, of that large section of the community which is tabulated as the criminal class is one on which it is most essential that just and right views should be held by the public, as the erroneous conclusions now entertained may do incalculable harm when used as a theme of discourse by Socialistic orators. They will constitute a somewhat long list when commented upon separately, but we will take first the nature and results of the separate system which Dickens denounced so vehemently, but which is now carefully and judiciously enforced in our English penal establishments. It is repeatedly stated and believed that this disciplinary system has so tremendous an effect on the prisoners that it shatters their nerves and breaks down their powers of endurance, so as to produce insanity in the majority of cases. This theory has lately been considered proved by the proceedings of one of the liberated Irish prisoners; but we deny absolutely that the instances of insanity which sometimes appear in prisons is the result of the separate system. So far as our practical experience goes, we have every reason to believe that only those prisoners become insane who have been predisposed to it from heredity or other physical tendencies before they ever saw the inside of a gaol, and who would have fallen victims to some form of mental disease under the most ordinary circumstances. Allowing that they may have been of sound mind previously, the complete reversal of all that has been life to them before is quite enough to fling undisciplined minds already disposed to such maladies into real or self-induced insanity, without the separate system being at all accountable for it.

The manner in which this disciplinary method is really enforced within our prisons is greatly misunderstood by outsiders beyond the walls. The object of it is twofold. Considered as a punishment it is certainly logical as well as salutary—that those who have committed crimes against their fellow creatures, and have injured them in their persons or their property, should be deprived of the privilege of intercourse with them, but even looked upon in that light it is not by any means the dumb, rigid solitude which the public suppose it to be. Prisoners can, and do, communicate with those set in authority over them, and however it may have been in times past we are glad to bear testimony to the high character and humane dispositions for which the officials carefully selected by the Prison Commissioners are almost universally distinguished. Besides the officers in charge, the chaplain has unlimited access to the cells, and can converse with their inmates as much as he pleases. There are also in many prisons appointed visitors from without, who at stated times, and under strict regulations, are allowed to see the prisoners with a view to their mental and moral improvement, and with the object also of assisting them to enter on a better mode of life when their sentence expires. It is most desirable that this system of prison visitation should be greatly extended. At present it is almost quite confined to the appointment of lady visitors to the female prisoners, but it might be of great use if it were extended to the male convicts, and, as regards both classes, much more extensively than it is at present. It will be seen from these facts that the separate system is not the cruel and dangerous punishment it is commonly supposed to be. There are, indeed, many religious orders whose rules inflict a far more rigorous and silent existence on exceptionally saintly persons than our worst criminals are ever made to endure. We have said that the object of this system is twofold, and its purpose, apart from the punishment of offenders, is to prevent all intercourse between the prisoners themselves, so that there may be no risk of the contamination of the less evilly-disposed by those more deeply tainted with crime. And here we touch another totally false impression as to prison life existing in the public mind. With the strange inconsistency which is apt to characterise many popular prejudices, the public, who resent so strongly the infliction of solitary confinement as a punishment, have a rooted conviction that, if a young or less heinous criminal is sent to prison, it is inevitable that he will be corrupted by older offenders, and actually instructed as to the mode of entering upon a course of graver guilt by professional criminals. We shall be able to demonstrate the complete fallacy of this most persistent idea, but the requirements of space oblige us to reserve our proofs for another occasion.

